# Neuromuscular transmission deficits in patients with CMT and ClC‐1 inhibition in CMT animal models

**DOI:** 10.1002/acn3.52252

**Published:** 2024-12-13

**Authors:** Thomas Skjærlund Grønnebæk, Helga Haahr‐Lillevang, Martin Skov, Kristina Kelly, Nathan R. Kerr, Jose A. Viteri, Andrea Jaworek, Amy Bartlett, Jane Bold, John Hutchison, Jorge Quiroz, Hatice Tankisi, Thomas Holm Pedersen, Henning Andersen, William David Arnold

**Affiliations:** ^1^ NMD Pharma A/S Aarhus Denmark; ^2^ Department of Neurology Aarhus University Hospital Aarhus Denmark; ^3^ NextGen Precision Health University of Missouri Columbia Missouri USA; ^4^ Department of Physical Medicine and Rehabilitation University of Missouri Columbia Missouri USA; ^5^ Department of Neurology The Ohio State University Columbus OH USA; ^6^ Department of Biomedicine Aarhus University Aarhus Denmark

## Abstract

**Objective:**

Charcot–Marie Tooth (CMT) is a hereditary neuropathy characterized by muscle weakness and fatigue with no approved therapies. Preclinical studies implicate neuromuscular junction (NMJ) transmission deficits in muscle dysfunction in CMT. This study aimed to evaluate NMJ function in patients with CMT types 1 and 2, and to determine whether enhancing NMJ transmission can improve muscle function in preclinical CMT models.

**Methods:**

First, an observational study involving single fiber electromyography (SFEMG) and clinical testing in patients with CMT 1 and 2 and healthy controls (HC) was conducted. Next, preclinical studies examined muscle function, specifically nerve‐stimulated muscle force after partially inhibiting ClC‐1 chloride channels with the novel small molecule NMD670.

**Results:**

Twenty‐one CMT patients (46.4 ± 14.4 years) and 10 HC (43.3 ± 12.7 years) were enrolled. SFEMG jitter (NMJ variability) was higher [median (range)] in the CMT patients [56 μs (35; 197 μs)] vs. HC [29 μs (19; 36 μs)], (*p* < 0.05). Blocking (NMJ failure) was higher in the CMT patients (13.4% (0.0; 90.9%)) vs. HC (0.0% (0.0; 4.5%)), (*p* < 0.05). In CMT, jitter and blocking correlated inversely with muscle strength, mobility, balance, and endurance. In CMT 1A and 2D mice, NMD670 increased both peak force and contractile endurance in vivo.

**Interpretation:**

Our study suggests that NMJ dysfunction contributes to muscle dysfunction in patients with CMT 1 and 2. Furthermore, our preclinical data provide proof‐of‐mechanism for recovery of muscle function with ClC‐1 inhibition in CMT mouse models. Collectively, these findings suggest that targeting NMJ dysfunction with ClC‐1 inhibitors could enhance muscle function in CMT patients, warranting further clinical trials.

## Introduction

Charcot–Marie Tooth (CMT) disease is a highly diverse group of hereditary peripheral neuropathies. CMT has an overall prevalence of ~1:2500 people,^1^ with CMT diagnosis is based on clinical assessment, nerve conduction studies, and genetic testing. Clinical features of CMT include motor signs and symptoms, such as muscle weakness, muscle atrophy, and fatigue, as well as sensory deficits that in combination cause substantial reduction in quality of life.^2^ CMT is broadly grouped into demyelinating (CMT 1) and axonal (CMT 2) types based on nerve conduction studies with conduction slowing being characteristic of CMT 1.^3^ With no approved medical therapies, treatment options are restricted to bracing, orthopedic surgery, and physiotherapy meaning leaving a substantial unmet medical for novel therapeutic approaches.

In general, symptoms in CMT occur and progress in a distal to proximal distribution referred to as “dying back,” typically attributed to length‐dependent axonal dysfunction or loss.^4^ At the distal, most vulnerable end of motor axons, the neuromuscular junction (NMJ) plays a vital role in muscle activation by transmitting electrical signals from motor neurons to muscle fibers. This is required for triggering muscle contraction and for securing muscle function. In CMT, muscle fibers can become denervated and their continued function rely on re‐innervation by neighboring motor neurons through collateral sprouting.^4^, ^5^ However, with collateral sprouting, NMJ transmission generally becomes less reliable, as seen in neurogenic disorders like amyotrophic lateral sclerosis and spinal muscular atrophy.^5^ Although this may be secondary or coexistent with motor neuron dysfunction, the process of denervation and re‐innervation, leading to unreliable NMJ transmission, could significantly contribute to symptoms of weakness and fatigue.^6^, ^7^, ^8^, ^9^, ^10^ In support of this notion, preclinical and clinical studies directly implicate NMJ transmission failure in CMT^11^, ^12^, ^13^ and drugs that improve transmission have been suggested as candidates for treatment of muscle symptoms in CMT.

Recently, partial inhibition of the skeletal muscle ClC‐1 chloride channel with a novel small molecule ClC‐1 inhibitor (NMD670) has been shown to enhance NMJ transmission and improve symptoms in animal models and patients with myasthenia gravis (MG).^14^ ClC‐1 is a skeletal muscle specific chloride channel that belongs to a family of 9 known ClC protiens in humans. The physiological role of ClC‐1 is to stabilize the resting membrane potential and acute regulation of ClC‐1 during muscle activity has been shown to be a key regulator of muscle fiber excitability during intense muscle activity.^15^, ^16^ Thus, CLC‐1 inhibition has the potential to therapeutically improve CMT symptoms.

The aim of the clinical study was to determine the extent to which NMJ transmission dysfunction contributes to clinical symptoms in CMT1 and 2 patients, and whether partial inhibition with a NMD670 could improve muscle force production in animal models of CMT1 and 2. A separate secondary aim of the clinical study was to investigate test–retest reliability and tolerability of clinical endpoints to inform design and power calculation of future clinical trials.

## Materials and Methods

### Clinical study design

**Figure 1:**
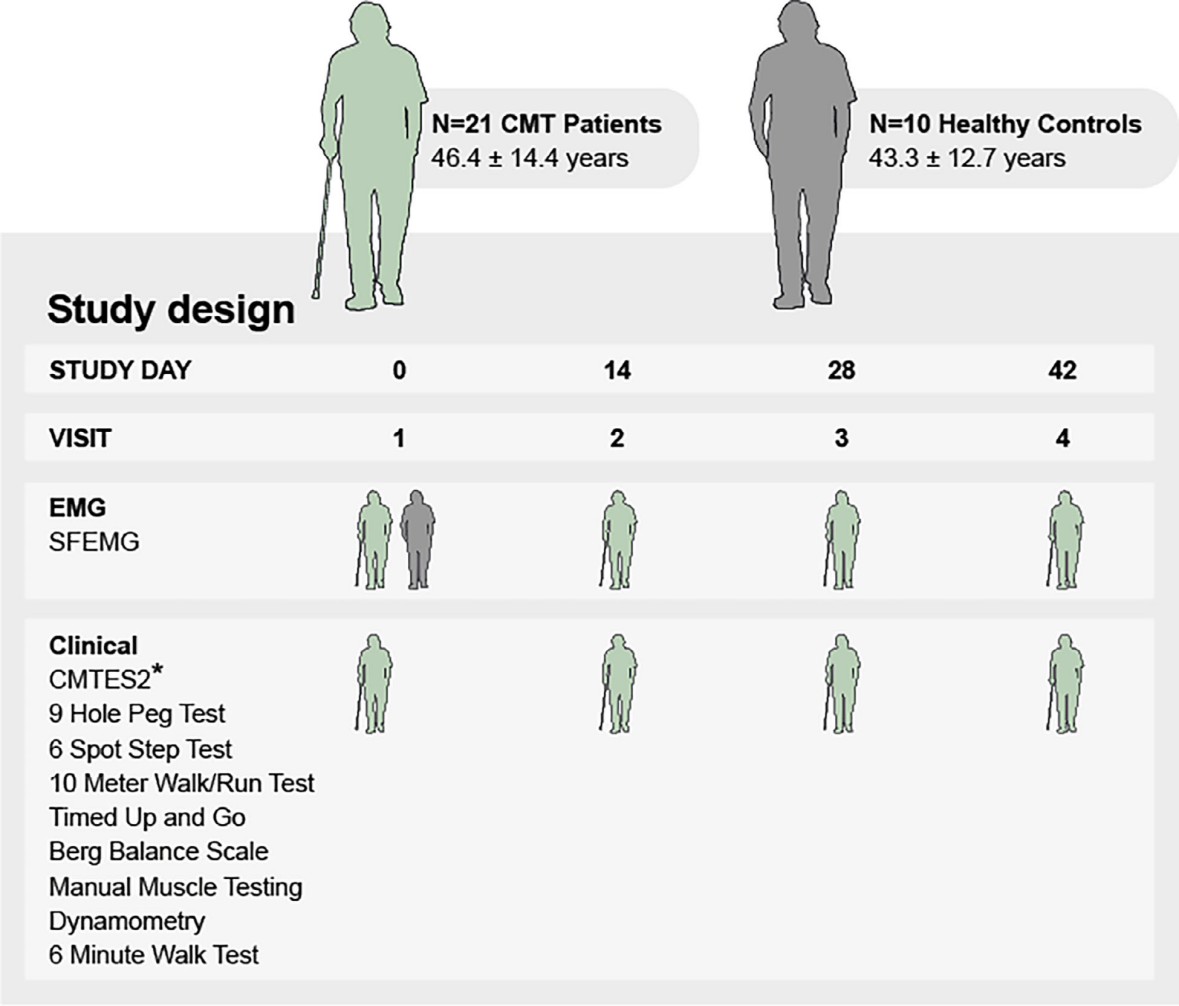
Clinical study design. Clinical tests are listed in the order of testing. CMTES2, CMT Examination Score2; EMG, electromyography; SFEMG, Single Fiber EMG. *CMTES2 only performed at visit 1.

This was a prospective, observational study designed to enroll patients with CMT1 and 2, and healthy controls (HC) with similar age at two study sites. The study protocol was reviewed and approved by The Ohio State University Wexner Medical Center Institutional Review Board and the Central Denmark region Committees on Health Research Ethics. All participants provided written informed consent before study participation. The CMT patients underwent four clinical evaluations on 4 separate days. After the baseline visit, the between visit interval was 2 weeks ±10 days (Fig. 1). Each visit involved clinical and electrophysiological testing. HC underwent electrophysiological testing at a single visit (Visit 1) (Fig. 1). The study was conducted in accordance with the Declaration of Helsinki and registered in the database Clinicaltrials.gov (NCT04980807). Reporting follows the STROBE guidelines where applicable.

### Participants

Study enrolment included patients with CMT1 and 2, and HC. The inclusion and exclusion criteria for the CMT patients and HC are listed in Table S1. The inclusion and exclusion criteria were defined to ensure certain CMT diagnosis, that the patients could complete the physical tests, and that their condition was stable.

### Study outcome measures

Outcome measures included single fiber electromyography (SFEMG) and functional assessments; isometric dynamometry, Manual Muscle Testing (MMT), 10 Meter Walk/Run Test (10MWT), Timed Up and Go (TUG), 6 min Walk Test (6MWT), Berg Balance Scale (BBS), 6 Spot Step Test (6SST), 9 Hole Peg Test (9HPT), and CMT Examination Score 2 (CMTES2). Detailed descriptions of functional assessments are given in the supplementary materials. CMT patients were permitted to use their typical bracing and/or assistive devices, which was documented at each visit. Adverse events (AEs) were collected throughout the study. Tolerability of outcome testing was evaluated in both HC (electrophysiological tests) and in the CMT patients (electrophysiological and clinical tests) at all study visits on a scale from 0 to 9 with 0 indicating no discomfort and 9 indicating worst possible discomfort.

### Single fiber electromyography

Voluntary SFEMG was performed on the anterior tibialis muscle using clinical electrodiagnostic systems (Ohio State University utilized Cadwell Sierra Wave [Kennewick, WA, USA] and Aarhus University Hospital utilized Keypoint.net [Natus, Middelton, WI, USA]) with concentric needle recording electrodes (25 mm × 30 gauge, Dantec) and 1–10 kHz for high‐ and low‐pass filter settings to record and analyze up to 22 apparent single muscle fiber action potential pairs during voluntary contraction.^17^ A total of 50–100 consecutive discharges were analyzed for each apparent single muscle fiber pair to determine parameters of jitter (mean consecutive delay as a measure of variability of NMJ transmission) and blocking (failure of NMJ transmission).^17^

### Statistical analysis of the clinical data

Statistical analyses were performed by an independent statistical consultancy group, Phastar, using SAS. Statistical coding was performed prior to database lock and in accordance with the statistical analysis plan for the study. For the primary aim of comparing NMJ transmission between CMT patients and HC, data that followed parametric distribution were analyzed with unpaired *t*‐tests. For data with unequal variances, Welch's test was used. For data that did not follow parametric distribution, a Mann–Whitney test was applied. For the analysis investigating associations between electrophysiological and clinical outcomes, Spearman correlation coefficients were calculated. For the test–retest reliability analysis, interclass correlation coefficient (ICC) was calculated. No corrections for multiple testing were performed due to the exploratory nature of the study.

### Preclinical study design and animal models

To represent CMT1 and CMT2, two mouse models were investigated using nerve‐stimulated muscle contractile function (*in vivo* plantarflexion torque and *in situ* force) at two study sites.

To model CMT1, hemizygous male and female B6.Cg‐Tg(PMP22)C3Fbas/J also known as the PMP22‐C3 mice (model of CMT1A) were obtained genotyped from the Jackson lab and included in the interventional study at 6–7 months of age.^18^ Animals were group‐housed with two individuals per cage, at 20–23°C at 55% humidity, on corncob bedding with at a 12:12 h light/dark cycle. Free access to water and chow (fed *ad libitum*). All experimental procedures were reviewed and approved by the Animal Care and Use Committee of the University of Missouri (protocol number 39905).

To model CMT2, a mouse model of CMT2D “CAST;B6‐*GarsNmf249*/JRwb” with JAX reference stock no. “17540” was used. The mouse was originally identified at the “Neuroscience Mutagenesis Facility” at The Jackson Laboratory in 2004. The P278KY mutation was originally isolated and described on an inbred C57BL/6J (B6) background, but later crossed onto a CAST background to increase the survival of the strain and resulted in the present model used here.^12^ Littermates without the P278KY mutation were used as wild type (WT) controls. All handling and use of animals complied with Danish Animal Welfare regulations, including euthanasia according to license 2017‐15‐0201‐01223, with appropriate extensions as of 2018.

### Assessment of in vivo muscle contractile function in CMT1A mice

Animals were sedated with inhaled isoflurane (3%–5% for induction, 1%–3% for maintenance) using a digital vaporizer (Somnosuite, Kent Scientific, Torrington, CT, USA). Plantarflexion torque following tibial nerve stimulation was assessed using an Aurora 1300A system and a footplate attached to a dual control torque motor (Aurora, ON, Canada) as previously described.^19^ The ankle plantarflexion angle was initially set at 90 degrees and then adjusted as needed to ensure consistent resting tension during repeated contractility testing for each animal.

To elicit torque responses detected by the footplate system, the tibial branch of the sciatic nerve was stimulated by two inserted electrodes on either side of the tibial nerve. To optimize positioning of the stimulation electrodes, test pulses of low current amplitude were applied until a stable reproducible torque measurement was achieved during two consecutive stimuli. Current amplitude was then increased to ensure supramaximal stimulation. Then, a series of 1 s long trains of electrical stimulations (duration 0.2 ms per stimulation/pulse) were initiated with fixed frequencies of 1, 15, 30, 60, 120, and 200 Hz. The protocol included rest periods of 5, 10, 30, 60, and 120 s, between the stimulations, respectively. Torque was measured before and after administration of NMD670 or vehicle and followed by two analyses; (1) maximal torque was determined from each trace at each stimulation frequency, and (2) the ability to sustain torque production at 60, 120, and 200 Hz was analyzed by comparing torque 150 ms after initiating the nerve stimulation to the torque 50 ms before the stimulation was ceased. The decline in torque during the contraction as captured by this ratio is referred to as fade. The ratios of produced torque at these two timepoints (last 50 ms/first 150 ms) were calculated and the change (in % point) in this ratio from before test article intervention, to the recordings after intervention was calculated for each stimulation frequency.

### Administration of NMD670 and vehicle in CMT1A mice

After a baseline torque measurement had been completed using the foot‐pedal system, the test article was administered intraperitoneally; either NMD670 (10 mg/kg, in PBS) or vehicle (same volume of PBS) to the sedated mice. Then, 15 min was allowed to elapse before torque measurement was repeated. The dose of NMD670 was chosen based on previous work in other animal models with NMJ dysfunction.^14^ Animals were randomized into receiving NMD670 or vehicle, and the treatment was blinded for both experimenter and when analyzing the results. The study was a cross‐over study exposing all mice to two tests in the system allowing intra‐mouse comparisons between vehicle and NMD670 treatment. Mice were allowed 48 h of rest in the home cage before the experiment was repeated. A total of eight mice were included in the study.

### Assessment of in situ muscle contractile function in CMT2D mice

Mice were removed from their housing on the day of experiment and sedated with Isoflurane while placed on a heated mat, after which the Achilles tendon was blotted, tied by cotton string to a force transducer (Grass instruments) and cut distal to the cotton string, to allow recording of force from the lower limb during sciatic nerve stimulation. To perform stimulation of the sciatic nerve, two stimulation needle electrodes were inserted at the base of the tail, one on each side of the sciatic nerve. Optimal placement and supramaximal stimulation were ensured by applying short pulses via the stimulation electrodes and assessing the resulting force. When a stable response was obtained, the electrodes were secured with tape. After experiments ended, animals were terminated in sedation.

### Stimulation protocol CMT2D mice

After optimizing the electrode positioning for force recording, a stimulation protocol consisting of 10 contractions separated by 30 s (9 sets of twitch contractions, 1 tetanic contraction) was initiated and repeated throughout the experiment: During each of the first 9 sets of twitch contractions, 10 nerve stimulations were applied using 8–12 Volt pulses of 0.1 ms duration at 12 Hz. Then, a 1 s train of stimulation pulses was applied that alternated between 30 and 120 Hz to measure tetanic force at the two frequencies. Thus, once a tetanic contraction had been performed at either 30 or 120 Hz, the 9 sets of twitch contractions at 12 Hz trains were repeated before the next tetanic contraction was performed at the alternative stimulation frequency. When stable force production at 120 Hz had been observed during three consecutive measurements, NMD670 was administered intravenously (I.V.). Animals were monitored for 6 h after NMD670 administration during which the protocol was continuously running.

Force production was digitized with 1 kHz sampling rate using an AD converter (Cambridge Electronic Designs, UK) and analyzed in “Signal 6” (Cambridge Electronic Designs, UK). To analyze the force traces, the peak amplitude of each twitch peak at 12 and 30 Hz was measured, and the resting force subtracted. The tetanic force at 30 and 120 Hz was also quantified as a force‐time integral, or area under the curve (AUC) in g*s.

### Administration of NMD670 in CMT2D and WT

NMD670 was administered I.V once to each animal. For administration, NMD670 was dissolved in phosphate buffered saline at 2 mg/mL and dosed at 8 mg/kg in the sedated mice, both WT and CMT2D mice. The dose of NMD670 was chosen based on previous work in other animal models with NMJ dysfunction.^14^ These experiments were terminal.

### Preclinical plasma concentration of NMD670

Samples were collected in EDTA coated tubes for centrifugation at 10.000 g at 4°C (40°F), for 5 min to obtain 20 μL plasma. Plasma concentrations of NMD670 were determined by protein precipitation and liquid chromatography with mass spectrometric detection (LC–MS). NMD670 was used to prepare a 1 mg/mL solution in DMSO, adjusted for salt, which was then diluted to generate calibration spiking solutions (12.5, 25.0, 125.0, 250, 1250, 2500, 5000, 25,000, and 50,000 ng/mL) in DMSO from the primary stock solution. The resultant blank tissues were used for matrix calibration standards, which were prepared on ice on the same day as analysis was performed at 25.0, 50.0, 250, 500, 2500, 5000, 10,000, 50,000, and 100,000 ng/mL by spiking blank plasma and 2:1 with NMD670 spiking solution. The analysis for each sample was performed on an LC–MS system: Thermo Scientific™ Q Exactive™ Focus Orbitrap with a HESI‐II electrospray source and UHPLC system using a Phenomenex Luna Omega C18 50 mm × 2.1 mm analytical column (EMD Millipore) with a 1.6‐μm pore size at 60°C. An injection volume of 4 μL was used for all samples and standards with a flow rate of 0.8 mL/min. Mass spectrometry data were generated with negative electrospray ionization (ESI‐) in full scan (150–10,000 Da, 35000 resolution). This method was used for samples from both CMT1A and CMT2D mice.

### Statistical analysis of preclinical data

Normality of the data was checked by visually inspecting a QQ plot of the data. Data from CMT1A mice were analyzed using a two‐way ANOVA with the Tukey‐post hoc test.

Data from CMT2D mice were analyzed with paired *t*‐tests without correction for multiple comparison. Statistical analysis was performed with Graph Pad Prism version 10.2.2. An alpha level of 0.05 was regarded as significant.

## Results

### Study participants, demographics, baseline characteristics, and adverse events

A total of 23 CMT patients (11 in Aarhus and 12 in Ohio) and 10 HC (5 at each site) were screened for participation. From the total of 21 CMT patients enrolled, 18 completed all visits, two dropped out after visit 3, and one withdrew consent before first visit; all available data are included in the analyses. Ten HC were enrolled; all HC completed all assessments at the single visit. Demographic and baseline characteristics are presented in Table 1. No AEs were reported for HC. A total of 4 AEs were reported in the CMT patients: 1 fell during 6MWT—graded mild, 1 fell during 10MWT—graded mild, 1 experienced knee pain following isometric dynamometry—graded moderate, and 1 had a brief syncope, graded mild, with rapid recovery.

**Table 1:** Demographics and clinical baseline characteristics.

	HC	CMT	CMT1	CMT2
*N*	10	21	16	5
Age (years)	43.3 ± 12.7	46.4 ± 14.4	43 ± 11.7	56 ± 19.3
Gender (M/F)	4/6	5/16	3/13	2/3
Charcot–Marie‐Tooth Examination Score 2 (CMTES2) total score	NA	10.1 ± 3.2 *N* = 20	10.3 ± 2.7 *N* = 15	9.4 ± 3.4 *N* = 5
9 Hole Peg Test (s)	NA	21.6 ± 4.7 *N* = 20	21.9 ± 5.3 *N* = 15	21.0 ± 2.2 *N* = 5
6 Spot Step Test (s)	NA	9.9 ± 2.6 *N* = 20	9.5 ± 2.7 *N* = 15	10.9 ± 1.8 *N* = 5
10 meter walk/run test (s)	NA	6.1 ± 2.1 *N* = 20	5.8 ± 1.8 *N* = 15	6.9 ± 3.1 *N* = 5
Timed up and go (s)	NA	7.9 ± 1.9 *N* = 20	7.4 ± 1.6 *N* = 15	9.4 ± 2.2 *N* = 5
Berg balance scale total score	NA	51.3 ± 5.0 *N* = 20	52.1 ± 4.8 *N* = 15	49.0 ± 5.6 *N* = 5
Manual muscle testing total score	NA	249.6 ± 17.3 *N* = 19	251.2 ± 15.6 *N* = 14	245.2 ± 23.0 *N* = 5
Non‐dominant ankle dorsi flexor strength—Aarhus (Nm)	NA	12.0 ± 4.5 *N* = 9	12.1 ± 4.8 *N* = 8	11.3 *N* = 1
Right ankle dorsi flexor strength—Ohio (*N*)	NA	95.9 ± 58.9 *N* = 7	96.9 ± 65.5 *N* = 4	94.6 ± 62.9 *N* = 3
6 min walk test total distance (m)	NA	455.3 ± 102.8 *N* = 20	464.9 ± 93.7 *N* = 15	426.7 ± 134.8 *N* = 5
6 min walk test 6/1 min (%)	NA	−6.6 ± 13.9 *N* = 20	−5.2 ± 14.7 *N* = 15	−10.8 ± 11.2 *N* = 5

Data are mean ± SD.

### CMT patients demonstrate notable NMJ transmission deficits compared to HC

**Figure 2:**
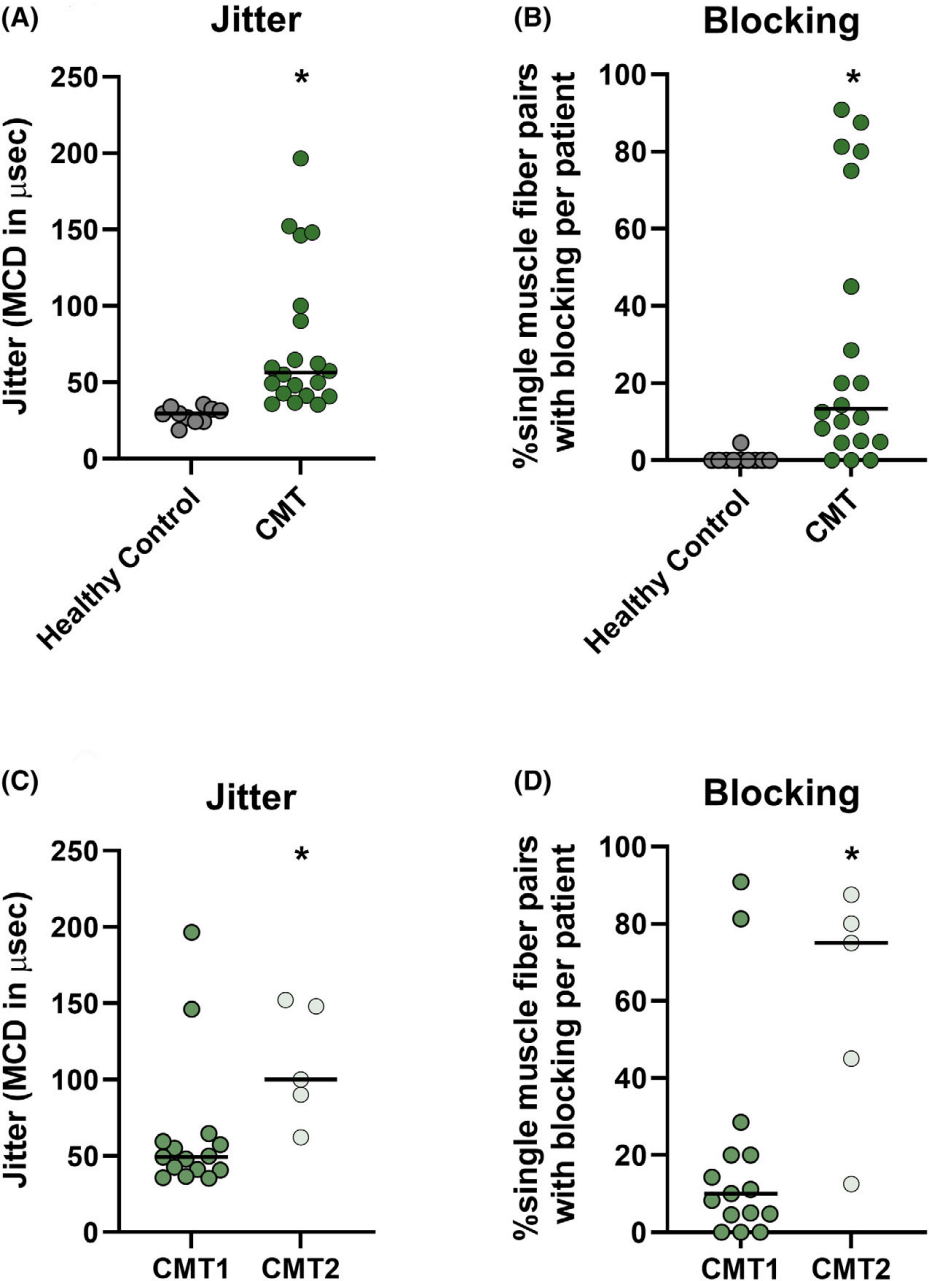
Observations of significant NMJ transmission deficits in CMT1 and 2 patients as evaluated on SFEMG. Jitter and blocking from visit 1 shown as individual data with medians for HC and CMT participants (A, B), and when stratified for the CMT1 and CMT2 patients (C, D). For jitter, each dot represents the median jitter of up to 22 single muscle fiber action potential pairs for 1 participant. For blocking, each dot represents % blocking calculated by dividing the number of blocking events with the total number of single muscle fiber action potential pairs for the particular participant. MCD, mean consecutive difference. * Statistically significantly different (*p* < 0.05).

For the primary aim, SFEMG jitter and blocking were compared between the CMT patients and HC using data from study visit 1 (baseline). Median SFEMG jitter, a measure of NMJ transmission variability, was increased by >90% in the CMT patients compared to HC [CMT: median jitter 56 μs (range: 35; 197) versus HC: median 29 μs (range: 19; 36), *p* < 0.01] (Fig. 2A). Similarly, SFEMG blocking, a measure of NMJ transmission failure, was increased in CMT patients versus HC [CMT: median blocking 13.39% (range: 0; 90.9) versus HC: median blocking 0% (range: 0; 4.5), *p* < 0.01].

In addition to comparing SFEMG between the CMT patients and HC, the SFEMG jitter was also compared between the CMT1 and CMT2 patients. While jitter was observed to be higher in the CMT2 patients, it should be noted that there were only five CMT2 patients enrolled in the study [CMT2: median jitter 100 μs (range: 62; 152) versus CMT1: median jitter 493 μs (range: 35; 197), *p* < 0.05] (Fig. 2C). SFEMG blocking was also increased in CMT2 versus CMT1 patients [CMT2: median blocking 75.0% (range: 12.5; 87.5) versus CMT1: median blocking 10.0% (range: 0.0; 90.9), *p* < 0.05] (Fig. 2D).

### NMJ transmission deficits correlate with symptoms in CMT patients

Associations between electrophysiological and clinical endpoints are summarized in Table 2. The performance on clinical parameters related to strength, balance, and mobility were inversely associated with both jitter and blocking. The SSST, 10MWT, and TUG were positively associated with jitter and blocking indicating that longer time to complete these tasks (worse performance) are associated with higher levels of jitter and blocking (worse NMJ transmission). For isometric ankle dorsi flexor strength, the total score of the BBS, and 6MWT were all negatively associated with jitter and blocking indicating that lower performance in these tasks were associated with higher degree of NMJ dysfunction. CMTES2, MMT, and 9HPT were not associated with jitter and blocking in the tibialis anterior.

**Table 2:** Associations between SFEMG parameters and clinical outcomes.

Clinical outcome measure	Association with Jitter	Association with Blocking
Right ankle dorsi flexor strength—Ohio	−0.89 (*p* < 0.05)	−0.75 (*p* = 0.05)
Non‐dominant ankle dorsi flexor strength—Aarhus	−0.78 (*p* < 0.05)	−0.63 (*p* = 0.07)
6 spot step test	0.67 (*p* < 0.01)	0.533 (*p* < 0.05)
Berg balance scale total score	−0.61 (*p* < 0.01)	−0.55 (*p* < 0.05)
Timed up and go	0.61 (*p* < 0.05)	0.46 (*p* < 0.05)
6 min walk test total distance	−0.59 (*p* < 0.05)	−0.48 (*p* < 0.05)
10 meter walk/run test	0.54 (*p* < 0.05)	0.33 (*p* = 0.14)
6 min walk test 6th/1st minute	−0.47 (*p* < 0.05)	−0.62 (*p* < 0.01)
9 Hole Peg Test	0.28 (*p* = 0.23)	0.02 (*p* = 0.95)
Manual muscle testing total score	−0.19 (*p* = 0.44)	−0.07 (*p* = 0.78)
CMTES2 total score	0.08 (*p* = 0.73)	0.02 (*p* = 0.94)

Data are Spearman's correlations and *p*‐values. Isometric dynamometry was performed using different procedures at Aarhus and Ohio sites as noted in the methods, and hence, these data were analyzed separately by site.

### Test–retest reliability and tolerability of SFEMG and clinical tests

Test–retest reliability was assessed for SFEMG and clinical outcomes by calculating ICC values based on data from study visits 1–4 from the CMT patients. Test–retest reliability estimates are summarized in Table S2. Tolerability was evaluated on a scale from 0 to 9 with 0 indicating no discomfort and 9 indicating worst possible discomfort. Tolerability scores for the different procedures at each visit for the CMT patients and HC are summarized in Table S3. In general, the assessments were well tolerated.

### Effect of ClC‐1 inhibition with NMD670 in CMT1A mice

**Figure 3:**
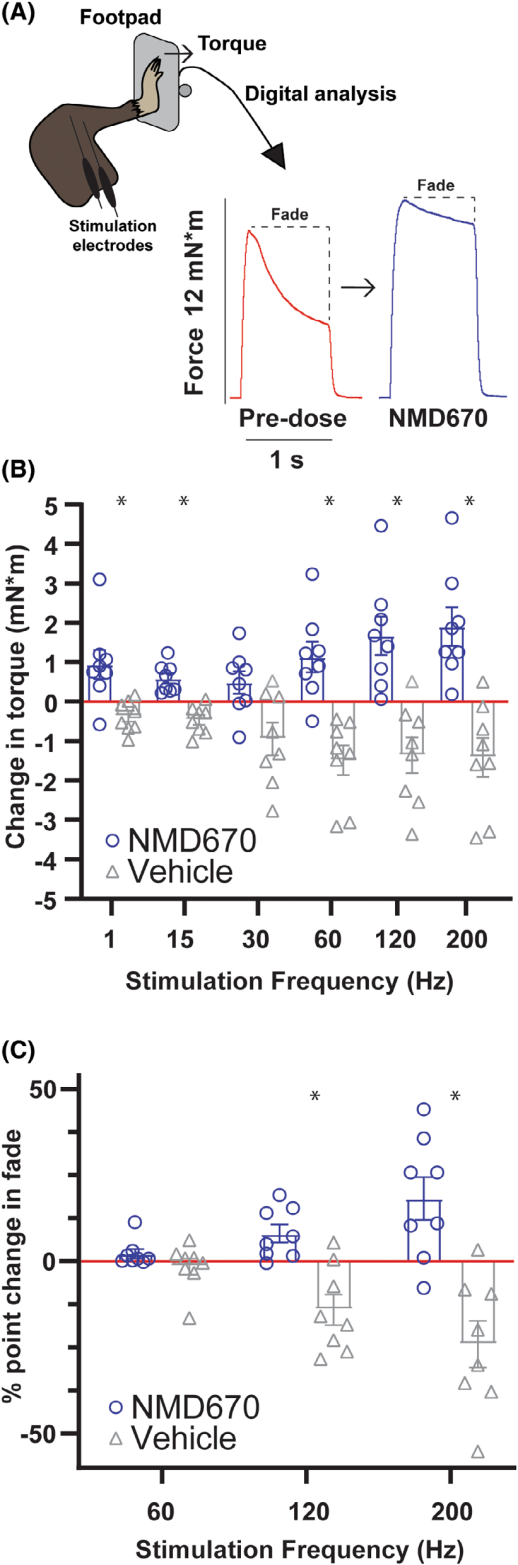
Effect of NMD670 on muscle force in CMT1A mice. (A) Schematic of experimental set‐up and representative force traces (torque, in mN*m) from CMT1A mice, at 200 Hz (stimulated for 1 s), before (red traces), and after receiving NMD670 (blue traces). (B) Change in force (torque, in mN*m) from before to after administration of either Vehicle (gray) or NMD670 (blue), during tibial nerve stimulation at each stimulation frequency. Blue = NMD670, gray = vehicle, *n* = 8 in both groups. (C) Change in force ratio between initial force at 150 ms after initiating stimulation and force at end of stimulation for frequencies at 60, 120, and 200 Hz, before and after administration, for each individual mouse. Blue = NMD670, gray = vehicle, *n* = 8 in both groups. Box plots overlays show means ± SEM. Blue = NMD670, gray = vehicle. * Statistically significantly different (*p* < 0.05).

The experimental approach and representative torque traces at 200 Hz from a CMT1A mouse before and after NMD670 administration are shown in Figure 3A. Peak muscle torque increased in the CMT1A mice when receiving NDM670. Such recovery of torque was generally observed at all stimulation frequencies explored and contrasted observations of reduced torque when vehicle was administered (considered a time effect). Thus, at 60 Hz, average torque increased 1.13 mNm when the mice received NMD670, while it declined 1.49 mNm when they received vehicle (Fig. 3B). Similarly at 120 Hz, torque increased by 1.67 mNm with NMD670 and decreased by 1.36 mNm with vehicle. This difference between NMD670 and vehicle was statistically significant at all tested stimulation frequencies (*p* < 0.05), except at 30 Hz (*p* = 0.0815). The ability to sustain plantarflexion torque during stimulated contraction was also improved when mice were administered NMD670 compared to vehicle (Fig. 3C). Thus, the ratio of peak torque at the last 50 ms over force at the first 150 ms (last 50 ms/first 150 ms) was improved by NMD670 at all tested frequencies in the CMT1A mice but declined when receiving vehicle. Finally, at 200 Hz, the maintenance of torque improved ~18% when receiving NMD670 but decreased by ~24% when receiving vehicle (Fig. 3C), This difference was statistically significantly different (*p* < 0.01) at stimulation frequencies of 120 and 200 Hz.

### Effect of ClC‐1 inhibition with NMD670 in CMT2D mice

**Figure 4:**
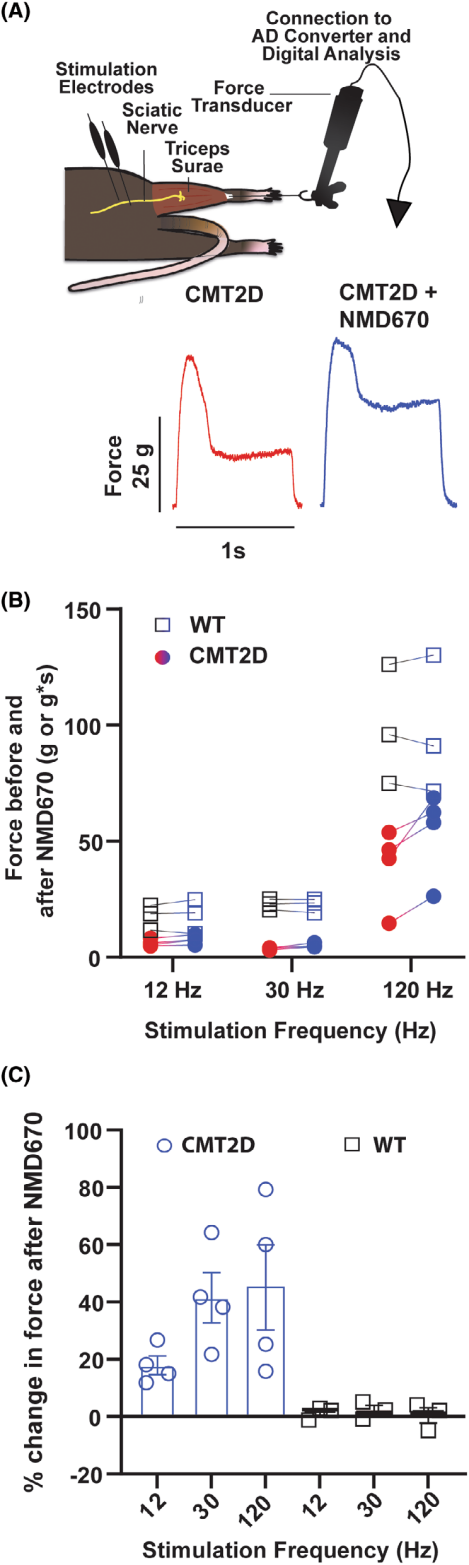
Effect of NMD670 on muscle force in CMT2D mice. (A) Schematic of experimental set‐up and representative force traces (force, in gram) from CMT2D mouse at 120 Hz, (stimulated for 1 s), before (red trace), and after administration of NMD670 (blue trace). (B) Absolute force in grams for 12 Hz and 30 Hz stimulation, and area of force trace for 120 Hz (g*s) before and after administration of NMD670 (red to blue circles) in 4 CMT2D mice and 3 WT mice (black to blue squares). (C) Change in stimulated force in % from before to after administration of NMD670, during sciatic nerve stimulation at each stimulation frequency from CMT2D mice (blue) and WT mice (black squares) shown in (B).

The experimental approach and representative force traces from a CMT2D mouse before and after NMD670 administration are shown in Figure 4A. As can be seen, the force traces showed substantial fade during the contraction. When NMD670 was administered, there was an increase in peak force and force integral, but there was a remaining fade after administration.

Generally, the CMT2D mice generated lower absolute force when compared to WT mice and they had lower peak twitch force relative to body weight (Fig. 4B, Table 3). Compared to WT mice, the CMT2D mice also displayed inability to maintain tetanic force and twitch force during repeated stimulations: During 10 nerve stimulations at 12 Hz, the ratios between the 4^th^ (T4) or 10^th^ (T10) peak twitch force to the 1^st^ (T1) peak twitch force declined by 12% and 18% in CMT2D mice, respectively, whereas in WT mice a decline larger than 6% was not observed (Table 3).

**Table 3:** Muscle force from CMT2D and WT mice before, and after administration of NMD670.

Force	WT mice		CMT2D mice	
	Before NMD670	After NMD670	Before	After NMD670*
Twitch force (g)	21.6 ± 1.3	22.2 ± 2.3	6.18 ± 0.73	7.5 ± 0.92*
Twitch force relative to Body Weight	0.98 ± 0.08	1.02 ± 0.12	0.42 ± 0.05	0.51 ± 0.05*
T4/T1	96.6 ± 1.3	97.7 ± 06	88.2 ± 5.1	97.8 ± 5.0*
T10/T1	97.3 ± 0.6	99.2 ± 0.3	82.0 ± 6.0	93.8 ± 5.7*
120 Hz AUC (% of initial)	100	100.7 ± 2.7	100	145.1 ± 14.8*
30 Hz AUC (% of initial)	100	102.9 ± 1.9	100	141.5 ± 8.75*

* “After NMD670” in the CMT2D colum indicates that all effects were statistically significantly different (*p* < 0.05).

In all CMT2D mice, NMD670 administration (8 mg/kg) increased in absolute twitch and tetanic force (Fig. 4B, Table 3), and it improved relative force peaks (Fig. 4C, Table 3). The effect of NMD670 was observed for all measured parameters, with normalization of T4/T1 and T10/T1 ratios of twitch force, and a 25% increase in absolute twitch force. In CMT2D mice following NMD670 administration tetanic force increased by 41 and 45% during 30 and 120 Hz stimulation, respectively (Fig. 4C). The changes were statistically significantly different (*p* > 0.05), see Table 3 for details. In contrast, there were no significant changes in twitch or tetanic force in the WT mice after administration of NMD670.

### Plasma NMD670 exposure in CMT1A and 2D mice

To confirm dosing of CMT1A mice, a blood sample was drawn after each test day and analyzed for NMD670 content. The results are displayed in Table S4. There were no detectable levels of NMD670 in any mice designated to receive vehicle on either test day, while mice designated to receive NMD670 had levels ranging from 38,420 ng/mL (120 μM) to 83,554 ng/mL (260 μM) with mean total plasma concentration of 64,353 ng/mL (200 μM). Plasma samples were collected between 26 and 54 min (mean: 35.8 min) after dosing of test article.

Similarly, in CMT2D mice a blood sample was drawn after end of experiment, but not in WT mice. NMD670 were present at levels ranging from 29,643 ng/mL (92 μM) to 44,379 ng/mL (138 μM) with mean total plasma concentration of 36,390 ng/mL (113 μM). Plasma samples were collected between 80 and 197 min (mean: 137 min) after dosing of NMD670.

## Discussion

In this study, we first evaluated whether NMJ transmission deficits are evident in patients with CMT1 and 2, and whether the magnitudes of these deficits were associated with symptom severity. Using SFEMG, we report notable NMJ transmission dysfunction that correlated with symptoms of weakness, fatigue, and impaired balance and mobility in 21 CMT1 and CMT2 patients. As a preclinical proof‐of‐concept, we also investigated modulation of NMJ transmission as a method to improve muscle dysfunction and demonstrate that treatment with a ClC‐1 inhibitor (NMD670) increased muscle force production in mouse models of both CMT1 and CMT2. This study highlights the role of NMJ dysfunction in CMT and supports the future clinical development of ClC‐1 inhibitors, such as NMD670, for patients with CMT.

Previous preclinical and clinical studies have reported altered NMJ structure and indicated NMJ dysfunction in selected CMT genotypes.^11^, ^13^ The findings of our clinical observational study extend these findings by showing that NMJ transmission deficits are a common feature across CMT patients with different genotypes. The significant correlations between NMJ transmission deficits and clinical assessments of function further implicate NMJ failure in muscle dysfunction in patients with CMT.

Out of the 21 CMT patients, 19 (90%) presented with blocking on SFEMG indicating a high incidence of NMJ transmission dysfunction in CMT. In a study involving 97 patients with myasthenia gravis (MG), a prototypical neuromuscular junction (NMJ) disorder, blocking on SFEMG was observed in approximately 20% of the patients.^20^ This MG study assessed SFEMG in three muscles (frontalis, extensor digitorum, orbicularis oculi) and used similar concentric needle electrodes and filter settings to investigate NMJ dysfunction as in the present study.^20^ Therefore, the level of NMJ dysfunction observed in patients with CMT1 and CMT2, exceeded the findings of NMJ failure in MG patients, where motor symptoms are entirely caused by NMJ dysfunction. However, the relatively small sample size of the current observational study may impair the ability to directly compare findings to previous observations in MG. Still, the correlations between NMJ dysfunction and clinical symptoms observed in this study, combined with the evidence of NMJ transmission blocking in CMT, support the idea that NMJ transmission deficits may significantly contribute to CMT disease burden.

Prompted by these clinical observations, we conducted preclinical experiments with NMD670 in mouse models of both CMT1 and CMT2. The B6.Cg‐Tg(PMP22)C3Fbas/J also known as PMP22‐C3 mice (CMT1A) and the CAST;B6‐*GarsNmf249*/JRwb CMT2D mice were chosen as models of demyelinating and axonal forms of CMT, respectively. Previous studies have indicated NMJ abnormalities in these mice models.^12^, ^18^ In the CMT1A mice, the effect of NMD670 was tested on in vivo muscle torque in a cross‐over study where each animal received placebo and NMD670 in a randomized and blinded order with washout between trials. In the CMT2D mice, the effect of NMD670 was tested on in situ muscle force comparing the effect of NMD670 between CMT2D mice and WT mice. In both experimental designs and with both CMT animal models, NMD670 increased muscle function and improved the ability to sustain force. This was observed at all stimulation frequencies compared to the respective control (vehicle/placebo or WT). These data provide preclinical proof‐of‐mechanism for ClC‐1 inhibition as a novel therapeutic approach in CMT to recover muscle function and improve stamina.

In conclusion, the current study demonstrates that patients with CMT, regardless of genotype, demonstrate notable NMJ transmission dysfunction that correlates with disease severity. Treatment of mouse models of CMT1A and CMT2D with NMD670 improves muscle contractile function. Collectively, our study provides evidence for the concept of alleviating NMJ transmission dysfunction as a potential therapeutic approach to improve muscle function in patients with CMT and supports clinical development of NMD670 for the treatment of CMT.

## Author Contributions

H.H.L, T.S.G., M.B.S, K.K., T.H.P., and W.D.A. drafted the manuscript. All authors contributed to the design, carried out the study, and/or contributed to data analysis. All authors critically revised the manuscript and provided intellectual contributions.

## Fundin Information

The study was sponsored by NMD Pharma A/S to H.A. and W.D.A.

## Conflicts of Interests

The study was sponsored by NMD Pharma A/S to H.A. and W.D.A. H.H. received grant funding from CSL Behring. T.S.G., M.B.S, J.B., J.H., J.A.Q., and T.H.P. were or are employed by NMD Pharma A/S and may own and/or hold options/restricted stock units in the company. K.K. has received consulting fees from NMD Pharma. H.A. has received grant funding from CSL Behring and received consulting fees from Zealand Pharma. He has received payment or honoraria for lectures, presentations, speakers bureaus, manuscript writing or educational events from Argenx, UCB, and Alexion, and payment for participation on a Data Safety Monitoring Board or Advisory Board for Alexion, UCB, Amicus Pharmaceuticals, Lundbeck, NMD Pharma. W.D.A. has received grant funding from NMD Pharma and Avidity Biosciences, consulting fees from Avidity Biosciences, NMD Pharma, Dyne Therapeutics, Genentech, Design Therapeutics, Cadent Therapeutics, Catalyst Pharmaceuticals, and speaker honorarium from University of Rochester, travel support from NMD Pharma, and from Novartis for serving as Chair and member of Data Safety and Monitoring Board. All other authors have no competing interests.

## Supporting information


Data S1.

## Data Availability

The raw data files can be made available upon reasonable request. The raw data from the clinical study are not publicly available because the information could compromise the participants' privacy. These data can be provided by NMD Pharma A/S pending scientific review, a review of data privacy compliance, and a completed data transfer agreement. Requests for data should be submitted to NMD Pharma. Materials described here may be made available to qualified, academic, noncommercial researchers through a material transfer agreement upon contacting NMD Pharma A/S.
