# Supplementary material for: Neuromuscular transmission deficits in patients with CMT and ClC‐1 inhibition in CMT animal models
Source: Ann Clin Transl Neurol. 2024 Dec 13;12(2):320–31. doi: 10.1002/acn3.52252 (PMC11822784; doi:10.1002/acn3.52252)
Supplement: Supplementary file 1 — Data S1. [file ACN3-12-320-s001.docx]

**Neuromuscular Transmission Deficits in Patients with Charcot-Marie-Tooth Disease and ClC-1 Inhibition to Restore Muscle Function in Animal Models**

**Supplementary Material**

## Functional assessments

### Manual Muscle Testing (MMT)

The MMT was performed on 15 muscle groups, including shoulder abduction, elbow flexion/extension, wrist flexion/extension, long thumb flexors, hip flexors/extensors, hip abductors, knee flexors/extensors, ankle dorsiflexors/plantarflexors, and neck flexors/extensors. All but the neck muscle groups were tested bilaterally. Positioning and grading were standardized and applied as previously described^1, 2^. See Supplementary Table 1 for translation of the modified Medical Research Council (MRC) scale into the 0-10 ordinal scale that was used for analysis.

### Isometric Dynamometry

Isometric dynamometry was performed using each site’s respective available equipment: The Ohio State University utilized the Accurate Test of Limb Isometric Strength (ATLIS) chair, which is a fixed frame apparatus with an adjustable wireless loadcell. Ankle dorsiflexion was tested on the right side in an upright seated position as previously described^3^. Aarhus University Hospital utilized a Biodex Dynamometer, SYSTEM 3 PRO (Biodex Medical Systems, Inc.) and ankle dorsiflexion was tested on the non-dominant side, with the standardized positioning as previously described ^4^. A minimum of two trials were performed; additional trials were performed if there was greater than 15% variability between the first two assessments. Since different methods were used at the two sites two evaluate isometric dynamometry, the data collected at the two sites were analyzed separately.

### The 9 Hole Peg Test (9HPT)

The 9HPT is a timed test of finger dexterity and hand function. Participants take 9 pegs out of a reservoir, one at a time, place them into holes aligned in a 3-by-3 grid and then move them back to the reservoir again in the same manner. This had to be performed as fast as possible. The 9HPT is reliable and responsive in people with CMT^5-7^. Set-up and procedure were standardized as previously described ^8^. Two trials were performed with each hand; trials were averaged and used for analysis.

### The 6 Spot Step Test (SSST)

The SSST is a timed test of lower extremity function and balance. Participants walk in a standardized zig-zag pattern through a 1 x 5-meter course, sliding cylindrical blocks with their tested foot out of the course as they move through it ^9^. This test has been shown to be valid and reliable in neurodegenerative conditions and older adults ^10-12^; it has also shown responsiveness in polyneuropathy ^13^. Two trials were performed with each foot; trials were averaged and used for analysis.

### The 10 Meter Walk/Run Test (10MWT)

The 10MWT is a timed assessment meant to capture maximal walking or running performance over a short distance and is a reliable test in CMT^14^. Two attempts were performed and averaged for analysis.

### The Timed Up and Go (TUG)

The TUG is a timed mobility assessment involving rising from a chair, walking 3 meters at a comfortable pace, and returning to sit in the chair ^15^. TUG has been validated in neurologic and geriatric populations ^15, 16^. Two trials were performed and averaged for use in data analysis.

### The 6 Minute Walk Test (6MWT)

The 6MWT is a measure of endurance and aerobic capacity and can also provide an index of fatigability ^17^. Validity has been established in patients with CMT^18^. The test was administered according to the American Thoracic Society guidelines on a 30-meter course with standardized cues ^19^. A 10-minute seated rest was required prior to the start of the test. Total distance as well as percent change in distance walked between the first and sixth minutes were used for analyses.

### The Berg Balance Scale (BBS)

The BBS is a 14-item assessment of balance during functional tasks. Each item is scored on a 0 to 4 scale where higher scores indicate better performance. The total score used for analysis is the sum of the individual scores for each item with a maximum score of 56. It has demonstrated validity in CMT^20^. Items were administered as previously described ^21^.

### The CMT Examination Score (CMTES2)

The CMTES2 is a composite scoring system to assess sensory and motor impairment in CMT patients. It is a sub-score of the CMT Neuropathy Score. Each item is scored on a 0-4 ordinal scale where higher scores indicate greater severity of impairment with a maximum score of 28 ^22^.

| **Supplementary Table 1. Inclusion and exclusion criteria for CMT patients and HC participants** | |
| --- | --- |
| **CMT Participants** | |
| **Inclusion criteria** | **Exclusion criteria** |
| Age $\geq$18 years | Ulceration that would interfere with functional ability |
| Diagnosis of CMT Type 1 or 2 via   - genetic testing **or** - a combination of clinical presentation and electrodiagnostic testing | Severe deformity or ankle contracture that would sufficiently limit passive range of motion to affect assessment of dorsiflexion strength |
| Physical ability (all must apply)   - ambulate 10 meters without bracing - bilateral ankle plantarflexion strength between 2- and 5 (inclusive) on the Medical Research Council (MRC) scale - bilateral ankle dorsiflexion strength between 2- and 4+ (inclusive) on the MRC scale | Significant change in physical activity or exercise (e.g. significant in- or decrease in intensity or frequency) within 8 weeks before Study Day 1 or inability to maintain the baseline level of physical activity throughout the study |
| Ability to adhere to study schedule and procedures | Moderate to severe neuropathic or inflammatory/musculoskeletal pain that would interfere with performance of the tests |
| Stable concomitant medications for 2 months prior to enrolment | A diagnosis of a primary NMJ disorder such as myasthenia gravis, Lambert Eaton Myasthenic Syndrome or congenital myasthenic disorder |
| Ability to provide written informed consent | Any disability or condition that would prevent completion of the study tasks |
|  | Recent major surgery |
| **Healthy Control Participants** | |
| **Inclusion criteria** | **Exclusion criteria** |
| Age $\geq$18 years | Pregnant |
| Healthy male or female volunteers | Requiring prescription medicines likely to interfere with electromyography (EMG) recordings |
| Ability to provide written informed consent | Presence of current or previous medical condition which might interfere with participation in the study |

| Supplementary Table 2. Test-retest reliability estimates | | | | | | | | | |
| --- | --- | --- | --- | --- | --- | --- | --- | --- | --- |
| **Outcome Measure** | | **ICC** | | **Visit 1** | | **Visit 2** | | **Visit 3** | **Visit 4** |
| **Electrophysiological Outcomes** | | | | | | | | | |
| Jitter (μs) | 0.50 | | 75.6 ± 47.7 | | 76.2 ± 47.7 | | 66.3 ± 33.4 | | 71.2 ± 38.5 |
| Blocking (%) | 0.48 | | 29.9 ± 33.2 | | 32.0 ± 30.3 | | 25.2 ± 26.9 | | 23.8 ± 23.4 |
| **Clinical Outcomes** | | | | | | | | | |
| 10 Meter Walk/Run Test (s) | 0.97 | | 6.1 ± 2.1 | | 5.9 ± 2.2 | | 5.8 ±2.3 | | 5.5 ± 2.0 |
| 9 Hole Peg Test (s) | 0.92 | | 21.6 ± 4.7 | | 21.3 ± 5.2 | | 20.2 ± 5.0 | | 20.0 ± 3.0 |
| Right Ankle Dorsi Flexor Strength – Ohio (N) | 0.88 | | 95.9 ± 58.8 | | 83.7 ± 46.3 | | 93.4 ± 48.6 | | 100.8 ± 60.7 |
| Non-Dominant Ankle Dorsi Flexor Strength – Aarhus (Nm) | 0.87 | | 12.0 ± 4.5 | | 13.5 ± 4.3 | | 13.7 ± 4.1 | | 13.8 ± 5.8 |
| 6 Minute Walk Test Total Distance (m) | 0.81 | | 455.3 ± 102.9 | | 474.4 ± 101.0 | | 481.3 ± 103.9 | | 490.4 ±  90.8 |
| 6 Spot Step Test (s) | 0.79 | | 9.9 ± 2.6 | | 9.1 ± 2.8 | | 8.8 ± 2.6 | | 8.3 ± 2.5 |
| Timed Up and Go (s) | 0.76 | | 7.9 ± 1.9 | | 7.6 ± 1.8 | | 7.6 ± 1.9 | | 7.3 ± 1.6 |
| Berg Balance Scale | 0.72 | | 51.3 ± 5.1 | | 53.2 ±3.4 | | 53.9 ± 3.3 | | 54.0 ± 3.3 |
| Manual Muscle Testing | 0.69 | | 249.6 ± 17.3 | | 256.3 ± 12.4 | | 256.5 ± 13.2 | | 259.7 ± 10.2 |
| 6 Minute Walk Test 6/1 minute (%) | 0.52 | | -6.6 ± 13.9 | | -6.5 ± 12.5 | | -3.0 ± 7.7 | | -3.3 ± 6.0 |

**Supplementary Table 2. Test-retest reliability estimates.** Data presented as mean ± SD and ICC.

| Supplementary Table 3. Tolerability | | | | | |
| --- | --- | --- | --- | --- | --- |
| **Outcome Measures** | **HC Visit 1** | **CMT Visit 1** | **CMT Visit 2** | **CMT Visit 3** | **CMT Visit 4** |
| **Electrophysiological Outcomes** | | | | | |
| SFEMG | 2 (1-6) | 3.5 (0-6) | 2.5 (0-9) | 3 (0-9) | 2.5 (0-8) |
| **Clinical Outcomes** |  |  |  |  |  |
| CMTES2 | NA | 0 (0-2) | NA | NA | NA |
| 9 Hole Peg Test | NA | 0 (0-4) | 0 (0-4) | 0 (0-4) | 0 (0-4) |
| 6 Spot Step Test | NA | 0 (0-4) | 0 (0-4) | 0 (0-4) | 0 (0-4) |
| 10 Meter Walk/Run Test | NA | 0.5 (0-4) | 1 (0-4) | 1 (0-4) | 0 (0-4) |
| Timed Up and Go | NA | 0 (0-4) | 0 (0-4) | 0 (0-4) | 0 (0-4) |
| Berg Balance Scale | NA | 1 (0-7) | 0 (0-5) | 0 (0-6) | 0.5 (0-3) |
| Manual Muscle Testing | NA | 0.5 (0-5) | 1 (0-3) | 0 (0-4) | 1 (0-4) |
| Isometric Dynamometry | NA | 1.5 (0-5) | 2 (0-5) | 2 (0-4) | 0.5 (0-4) |
| 6 Minute Walk Test | NA | 2 (0-5) | 1 (0-6) | 1.5 (0-5) | 1 (0-5) |

**Supplementary Table 3.** Tolerability was evaluated on a scale from 0-9 (0= no discomfort; 9= worst possible discomfort). Date presented as median (range).

**Supplementary Table 4** Plasma levels of NMD670 in individual plasma samples CMT1A. First column is in ng/mL and second column in µM.

| Mouse number | NMD 670 ng/mL | NMD670 µM |
| --- | --- | --- |
| 1 | 65053 | 202.6 |
| 2 | 57161 | 178.0 |
| 3 | 73259 | 228.1 |
| 4 | 38420 | 119.6 |
| 5 | 66321 | 206.5 |
| 6 | 61173 | 190.5 |
| 7 | 83554 | 260.2 |
| 8 | 45358 | 141.2 |

1. Personius KE, Pandya S, King WM, Tawil R, McDermott MP. Facioscapulohumeral dystrophy natural history study: standardization of testing procedures and reliability of measurements. The FSH DY Group. Phys Ther 1994;74:253-263.

2. Kendall F. Muscle testing and function with posture and pain, 5th ed. Philadelphia: Lippincott Williams & Wilkins, 2005.

3. Andres PL, Skerry LM, Munsat TL, et al. Validation of a new strength measurement device for amyotrophic lateral sclerosis clinical trials. Muscle Nerve 2012;45:81-85.

4. Harbo T, Brincks J, Andersen H. Maximal isokinetic and isometric muscle strength of major muscle groups related to age, body mass, height, and sex in 178 healthy subjects. Eur J Appl Physiol 2012;112:267-275.

5. Svensson E, Häger-Ross C. Hand function in Charcot Marie Tooth: test retest reliability of some measurements. Clinical Rehabilitation 2006;20:896-908.

6. Solari A, Laurà M, Salsano E, Radice D, Pareyson D. Reliability of clinical outcome measures in Charcot-Marie-Tooth disease. Neuromuscular Disorders 2008;18:19-26.

7. Piscosquito G, Reilly MM, Schenone A, et al. Responsiveness of clinical outcome measures in Charcot−Marie−Tooth disease. European Journal of Neurology 2015;22:1556-1563.

8. Mathiowetz V, Weber K, Kashman N, Volland G. Adult Norms for the Nine Hole Peg Test of Finger Dexterity. The Occupational Therapy Journal of Research 1985;5:24-38.

9. Nieuwenhuis MM, Van Tongeren H, Sørensen PS, Ravnborg M. The six spot step test: a new measurement for walking ability in multiple sclerosis. Mult Scler 2006;12:495-500.

10. Sandroff BM, Motl RW, Sosnoff JJ, Pula JH. Further validation of the Six-Spot Step Test as a measure of ambulation in multiple sclerosis. Gait Posture 2015;41:222-227.

11. Brincks J, Callesen J, Dalgas U, Johnsen E. Test-retest reliability and limits of agreement of the Six-Spot Step Test in people with Parkinson's disease. Clin Rehabil 2019;33:285-292.

12. Kondori RE, Cetin SY, Erel S. The Validity and Reliability of the Six-Spot Step Test (SSST) in Older Adults. Topics in Geriatric Rehabilitation 2020;36.

13. Kreutzfeldt M, Jensen HB, Ravnborg M, Markvardsen LH, Andersen H, Sindrup SH. The six-spot-step test - a new method for monitoring walking ability in patients with chronic inflammatory polyneuropathy. J Peripher Nerv Syst 2017;22:131-138.

14. Bray P, Cornett KMD, Estilow T, et al. Reliability of the Charcot-Marie-Tooth functional outcome measure. J Peripher Nerv Syst 2020;25:288-291.

15. Podsiadlo D, Richardson S. The timed "Up & Go": a test of basic functional mobility for frail elderly persons. J Am Geriatr Soc 1991;39:142-148.

16. Dunaway S, Montes J, Garber CE, et al. Performance of the timed "up & go" test in spinal muscular atrophy. Muscle Nerve 2014;50:273-277.

17. Montes J, McDermott MP, Martens WB, et al. Six-Minute Walk Test demonstrates motor fatigue in spinal muscular atrophy. Neurology 2010;74:833-838.

18. Mori L, Signori A, Prada V, et al. Treadmill training in patients affected by Charcot-Marie-Tooth neuropathy: results of a multicenter, prospective, randomized, single-blind, controlled study. Eur J Neurol 2020;27:280-287.

19. ATS Statement. American Journal of Respiratory and Critical Care Medicine 2002;166:111-117.

20. Monti Bragadin M, Francini L, Bellone E, et al. Tinetti and Berg balance scales correlate with disability in hereditary peripheral neuropathies: a preliminary study. Eur J Phys Rehabil Med 2015;51:423-427.

21. Berg K, Wood-Dauphine S, Williams JI, Gayton D. Measuring balance in the elderly: preliminary development of an instrument. Physiotherapy Canada 1989;41:304-311.

22. Murphy SM, Herrmann DN, McDermott MP, et al. Reliability of the CMT neuropathy score (second version) in Charcot-Marie-Tooth disease. J Peripher Nerv Syst 2011;16:191-198.
